# Path Planning for Mobile Robot Based on Improved Bat Algorithm

**DOI:** 10.3390/s21134389

**Published:** 2021-06-26

**Authors:** Xin Yuan, Xinwei Yuan, Xiaohu Wang

**Affiliations:** College of Intelligent Systems Science and Engineering, Harbin Engineering University, Harbin 150001, China; yuanxin@hrbeu.edu.cn (X.Y.); treepig@hrbeu.edu.cn (X.W.)

**Keywords:** path planning, bat algorithm, dynamic window approach, logarithmic decreasing strategy, Cauchy disturbance

## Abstract

Bat algorithm has disadvantages of slow convergence rate, low convergence precision and weak stability. In this paper, we designed an improved bat algorithm with a logarithmic decreasing strategy and Cauchy disturbance. In order to meet the requirements of global optimal and dynamic obstacle avoidance in path planning for a mobile robot, we combined bat algorithm (BA) and dynamic window approach (DWA). An undirected weighted graph is constructed by setting virtual points, which provide path switch strategies for the robot. The simulation results show that the improved bat algorithm is better than the particle swarm optimization algorithm (PSO) and basic bat algorithm in terms of the optimal solution. Hybrid path planning methods can significantly reduce the path length compared with the dynamic window approach. Path switch strategy is proved effective in our simulations.

## 1. Introduction

Path planning means that the robot searches in a state space using the distance transform or heuristics to find the path with the lowest cost from the initial state to the target state according to a certain performance index [1,2]. Its essence is to obtain the optimal or feasible solution under multiple constraints. It is the application direction of autonomous mobile robots, and also a core problem of multi-agent systems (MAS) [3,4]. A good mobile robot path planning technology can not only save a lot of time but also reduce the wear and capital investment of mobile robots [5]. At present, the commonly used path planning methods can be divided into two types: traditional algorithm and intelligent algorithm. Traditional heuristic path planning algorithms mainly include A*algorithm [6], D*algorithm [7], Dijkstra [8], etc. These algorithms generally use path length as the only indicator. The diversity of results is insufficient, and these algorithms can easily fall into a local optimum due to excessive “Greedy”. For this reason, many bionic intelligence algorithms have been proposed and applied to the path planning of mobile robots. These algorithms usually do not rely on specific problems. The problem-solving process and final result are random on a certain level. This provides the possibility of obtaining the optimal solution. Bionic intelligent algorithms mainly include particle swarm optimization [9,10], ant colony optimization [11], firefly algorithm [12], genetic algorithm [13], artificial fish swarm algorithm [14] and so on. In intelligent optimization algorithms, we can design the fitness function according to actual needs. Evaluation of the path can not only rely on the length but also on other factors.

Bat algorithm (BA) is an effective method for searching the global optimal solution proposed by Xin-She Yang of Cambridge University in 2010. It was proposed to simulate bats sending and receiving ultrasonic waves to prey [15]. The main idea of BA is to generate a set of initial solutions randomly and then have it search for the optimal solution through several iterations. At the same time, the algorithm generates a new local solution by flying randomly near the optimal solution. This strategy strengthens the algorithm’s local search capabilities.

Considering that in the planning process, the speed and accuracy of graph-based path planning methods depend on the granularity of the search space, those approaches are not suitable for real-time application [16]. Intelligent algorithm planning path is also difficult to meet the real-time requirement. Some improved BAT algorithms have been applied to optimize PID parameters and artificial neural network models. Tang et al. introduced multi-swarm strategy and adaptive inertial weight strategy for multi-robot path planning to improve the diversity of bat populations. Simulation results prove that the algorithm has higher efficiency than the particle swarm algorithm [17]. In this paper, BA is used as a global path planning algorithm combined with a local path planning algorithm to complete path planning. The Cauchy disturbance is introduced into the local update formula of the BA to increase the diversity of the population without increasing the complexity of the algorithm. At the same time, a smoothing function is added to the population fitness function to improve the quality of search results.

To improve real-time performance, some studies have proposed local navigation algorithms. Dynamic-window approach is a well-known velocity space algorithm. This approach uses the evaluation functions such as safety and smoothness to determine the next movement [18,19].

This paper proposes a global dynamic path planning method combining BA and DWA. In order to enhance the optimization capability of BA, we import logarithmic decreasing strategy and Cauchy disturbance into BA. Moreover, we develop a path switch strategy to reduce the planning time. The flow of this paper is as follows: Section 2 introduces the basic BA and its improved methods that we propose; Section 3 introduces the principle of the dynamic window method; Section 4 introduces the simulation results of the hybrid path planning method.

## 2. Global Path Planning Based on Algorithm

### 2.1. Basic Bat Algorithm

To simplify the characteristics of bat echolocation, Xin-She Yang set the following ideal rules:

(1) All bats use the difference in sensory echoes to determine the difference between food and obstacles.
(2) Bats fly randomly at a speed $V_{i}$, position $x_{i}$, and a fixed frequency $f_{i}$ (or wavelength). They use different wavelengths λ, and loudness $A_{0}$, to search for prey.
(3) Although the loudness changes in different situations, it is assumed here that the loudness changes from a large positive number $A_{0}$ to a minimum value $A_{\min}$.

The updating formula of frequency, speed and position of each bat in the algorithm is as follows:

(1) $$f_{i}=f_{\min}+(f_{\max}-f_{\min})\beta$$

(2) $$V_{i}^{\left(k\right)}=V_{i}^{\left(k-1\right)}+(x_{*}-{x_{i}}^{\left(k-1\right)})f_{i}$$

(3) $$x_{i}^{\left(k\right)}=x_{i}^{\left(k-1\right)}+V_{i}^{\left(k\right)}$$

where $f_{i}$ represents the frequency of the ith bat, $f_{\max}$ and $f_{\min}$ represent the maximum and minimum frequencies. β is a random variable drawn from a uniform distribution. $x_{i}^{\left(k\right)}$ and $V_{i}^{\left(k\right)}$ represent the position and velocity at generation k. $x_{*}$ is the current best solution.

For a local search, once a solution is selected from the current best solutions, each bat will generate a new local solution according to the random walk rule:

(4) $$x_{new}=x_{old}+\varepsilon A^{\left(k\right)}$$

where ε∈[−1,1] is a random number; $A^{\left(k\right)}=<A_{i}^{\left(k\right)}>$ is the average loudness of all bats in the same time step.

The loudness $A_{i}$, and the pulse emission rate $\gamma_{i}$, are updated according to the following iterative process. When the bat finds the prey, the loudness will decrease, the pulse rate will increase, and the loudness will change with any convenient value. The updated formulas are as follows:

(5) $$A_{i}^{\left(k+1\right)}=\alpha A_{i}^{\left(k\right)},\gamma_{i}^{\left(k+1\right)}=\gamma_{i}^{0}[1-\exp(-\gamma k)]$$

where α and γ are constants. For any 0<α<1 and γ>0, there is:

$$A_{i}^{\left(k\right)}\to 0,\;\gamma_{i}^{\left(k+1\right)}\to \gamma_{i}^{0},\;\mathrm{as}\;k\to \infty .$$

### 2.2. Improved Bat Algorithm

#### 2.2.1. Logarithmic Decreasing Strategy

The bat algorithm searches for the best solution through the bat groups flying in the solution space. The speed of bats affects the convergence speed and accuracy of the algorithm. In terms of the speed update formula of bat algorithm, the speed of each bat is affected by the previous generation of bats. However, the velocity coefficient of the previous generation of bats in the formula is a constant, which greatly reduces the diversity of bat movement. Both the bat algorithm and the particle swarm algorithm are optimization algorithms that combine global searches and local searches and have a lot in common. In the initial phase, the algorithm performs a global search which requires that bats have a large speed and quickly spread throughout the solution space. In the later stage of the algorithm, the bat has found the area where the optimal solution is located. At this time, the lower speed can enhance the local search ability and improve the convergence speed and accuracy of the algorithm. Therefore, the bat algorithm can improve the performance of the algorithm by adding dynamic weights to the speed update formula. For particle swarm optimization (PSO) there has been a lot of research, mainly including linear decreasing inertia weight strategy, Gauss decreasing inertia weight strategy, random inertia weight strategy, chaos inertia weight strategy and so on.

Through the above analyses, this paper chooses a dynamic inertia weight based on logarithmic decreasing strategy. This inertial weight guarantees the randomness of the speed of each bat. At the beginning of the algorithm, the bat group will be distributed throughout the entire solution space, enhancing the ability to jump out of the local extreme value. In the later stage of the algorithm, the speed of individual bats decrease and a more detailed search is carried out. In this paper, the bat algorithm is improved by using the logarithmic decreasing strategy. The expression of the logarithmic decreasing strategy factor and the speed update formula of the improved algorithm are:

(6) $$\omega =\omega_{\max}-a\cdot (\omega_{\max}-\omega_{\min})\cdot \log_{MaxIter}k+b\cdot \sigma \cdot randn()$$

(7) $$V_{i}=\omega V_{i}^{\left(k-1\right)}+(x_{*}-x_{i}^{\left(k-1\right)})f_{i}$$

where *MaxIter* is the maximum number of iterations. α is the logarithmic control factor. β is the disturbance control factor. σ is the deviation degree of the inertia weight ω from its mean value.

#### 2.2.2. Cauchy Disturbance

In the local search of bat algorithm, the newly generated solution is the simple addition of the current optimal solution and loudness, which has a low utilization capacity for the optimal solution. Therefore, Cauchy disturbance is added to the current optimal solution to increase the diversity of the population and maximize the random searchability of the bat algorithm. In addition, the distribution of random numbers generated by the Cauchy distribution is larger than that of the Gaussian distribution, which is more suitable for bats to jump out of the local optimal trap. The probability density function of a one-dimensional Cauchy distribution is:

(8) $$f_{t}(x)=(1/\pi )\cdot k/(k^{2}+{\left\|x\right\|}_{2}^{2}),-\infty <x<+\infty$$

The improved local new solution formula is as follows:

(9) $$x_{new}=x_{old}+n\cdot C(|x_{old}|,1)\cdot rand+\varepsilon A^{\left(k\right)}$$

where n=c⋅(MaxIter−k)/MaxIter. *c* is the disturbance control factor.

#### 2.2.3. Environment Model and Fitness Function

In this paper, grid map is used to model the working environment of a mobile robot. We simplify the robot as a particle and expand the obstacles in the grid map with the size of the robot to ensure that the planned path is safe. There are obstacles in the black grid, and the robot is not allowed to pass through. The white grid is a free grid, and the robot can walk freely.

Designing a fitness function is the key to path planning; this affects the convergence of the algorithm. The path planning needs to meet two basic conditions, they are, it cannot collide with obstacles, and the path should be as short as possible, so the path length and penalty function should be taken as the fitness function. In addition, the smoothness of a path is also an important index to evaluate a path. Therefore, the fitness function in this paper should include the above three terms.

Suppose the path represented by an individual $P=\{p_{0},p_{1},\ldots ,p_{n}\}$, where $p_{0}$ and $p_{n}$ (which are marked as S and E in figures) represent the starting point and the ending point, respectively. The fitness function designed in this paper is as follows:

(1) The shortest path is required, so the path length, as a fitness function, is the sum of the line lengths between all adjacent path points, which can be expressed by the following formula:
  (10) $$L=\prod_{i=1}^{n-1}\sqrt{{\left(x_{i+1}-x_{i}\right)}^{2}+{\left(y_{i+1}-y_{i}\right)}^{2}}$$
  where $\left(x_{i},y_{i}\right)$,$\left(x_{i+1},y_{i+1}\right)$ represents the horizontal and vertical coordinates of path points $p_{i}$ and $p_{i+1}$ in the grid map.
(2) Penalty function: used to remove obstacles in the search process.
  (11) $$f_{2}=\varphi \cdot m_{ob}$$
  where φ is the coefficient with the obstacle, which is a large constant. $m_{ob}$ is the number of collisions with obstacles. When the path is a collision free safe path, this item is 0.
(3) Path smoothness function: the steering cost of the mobile robot when following the path. Since the robot usually decelerates when turning, the size of the path turning angle is closely related to the working time of the robot. The turning angle in the grid is 0.25π or 0.5π. The path smoothness function is calculated as follows:
  (12) $$f_{3}=\frac{m_{1}\pi }{4}+\frac{m_{2}\pi }{2}$$
  where $m_{1}$ is the number of turning angle equals to 0.25π in the path and $m_{2}$ is the number of turning angle equals to 0.5π in the path.

Based on the three path evaluation functions above, the final fitness function of bat algorithm is as follows:

(13) $$F=\eta \cdot f_{1}+\lambda \cdot f_{2}+\mu \cdot f_{3}$$

where η, λ, and μ are the weight coefficients of path length, safety and smoothness respectively, and the proportion can be changed according to actual needs.

## 3. Dynamic Window Approach

Dynamic window approach (DWA) is a local path planning method considering the dynamic performance of a robot proposed by Dieter Fox in 1997 [19]. Its search space is a dynamic window composed of the achievable speed in a short time interval. This method only considers the safety trajectory generated by the allowable speed. It aims to search for the reachable linear velocity v and angular velocity ω of the robot under dynamic constraints at each moment, and then simulates the trajectory generated by (v,ω) in a short time at that moment, according to the objective function. The best combination of linear velocity and angular velocity are selected to control the robot’s movement [20].

### 3.1. Kinematics Model

DWA simulates trajectories under different combinations of linear and angular velocities based on a kinematic model, where the first step is to establish a mobile robot’s motion model. It is assumed here that the time interval is short. The mobile robot moves at a constant speed along a straight line during the interval time, and cannot perform omnidirectional movement, that is, it can only advance and rotate. The displacement of the robot in a time interval can be obtained by:

(14) $$\Delta x=v\Delta t\cos(\theta_{t})$$

(15) $$\Delta y=v\Delta t\sin(\theta_{t})$$

thus, the trajectory of the robot in a time interval can be obtained by:

(16) $$x=x+v\Delta t\cos(\theta_{t})$$

(17) $$y=y+v\Delta t\sin(\theta_{t})$$

(18) $$\theta_{t}=\theta_{t}+w\Delta t$$

### 3.2. Robot Velocity Space

The next step is to select the speed of the robot so that the trajectory of the robot can be predicted. In the speed space, the robot can have an infinite number of groups (v,ω). However, due to the constraints of maximal and minimal speed constraints, acceleration constraints, and safety constraints, the speed can be limited to a certain range [20].

(1) Maximal and minimal speed constraints:

The robot’s speed is divided into linear velocity v and angular velocity ω. Both v and ω are subject to maximal and minimal velocity constraints. Let $V_{m}$ denote the maximum and minimum speed space of the robot, that is:

(19) $$V_{m}=\{(v,\omega )|v\in [v_{\min},v_{\max}]\wedge \omega \in [\omega_{\min},\omega_{\max}]\}$$

(2) Acceleration constraints:

Because different motors have different performances, the acceleration of the robot is also different, and the robot is constrained by the speed that can be reached in the next time interval. Let $V_{d}$ represent the speed space that the robot can reach, $v_{c}$ and $\omega_{c}$ represent the speed and angular velocity of the robot at the current moment. $a_{v}$ represents linear acceleration, and $a_{\omega }$ represents angular acceleration.

(20) $$V_{d}=\{(v,\omega )|v\in [v_{c}-a_{v}\Delta t,v_{c}+a_{v}\Delta t],\omega \in [\omega_{c}-a_{\omega }\Delta t,\omega_{c}+a_{\omega }\Delta t]\}$$

(3) Safety constraint:

To avoid collision between the robot and the obstacle and ensure the safety of the robot, the speed of the robot should be reduced to 0 while approaching the obstacle. Let $V_{a}$ denote the safe velocity space of the robot.

(21) $$V_{a}=\{(v,\omega )|v\leq \sqrt{2*dist(v,\omega )*v}\wedge \omega \leq \sqrt{2*dist(v,\omega )*\omega }\}$$

where dist(v,ω) is the distance between the trajectory corresponding to the speed (v,ω) and the nearest obstacle.

### 3.3. Evaluation Function

The last step of the dynamic window approach is to evaluate the predicted trajectory. Here the evaluation function is divided into three indicators: azimuth evaluation function, distance evaluation function, and speed evaluation function.

The azimuth in the azimuth evaluation function refers to the angle difference between the current orientation and the target point orientation when the robot reaches the end of the planned trajectory; it is expressed by $180^{\circ }-\theta$. The smaller the angle difference, the larger the evaluation function value.

The distance evaluation function indicates how close the trajectory end is to the obstacle, and it would be discarded if the trajectory intersects the obstacle.

The speed evaluation function is used to evaluate the current speed of the robot, and the speed indirectly affects the distance function of the robot.

The overall evaluation function is:

(22) G(ν,ω)=σ(αheading(ν,ω)+βdist(ν,ω)+γvel(ν,ω))

In the above formula, heading(v,ω) represents the azimuth evaluation function, dist(v,ω) represents the distance evaluation function, and vel(v,w) represents the speed evaluation function. α, β, γ are the weight coefficients of the three terms in G(v,ω) respectively. In order to make the trajectory smoother, we normalized the three evaluation functions separately, that is, divide each term by the sum of the three terms. σ is a smoothing function.

## 4. Fusion Algorithm and Path Switching Strategy

### 4.1. Fusion Method

The dynamic window approach is to plan a path in real time according to the local environment information, so that it has a good obstacle avoidance ability. However, this method does not consider the problem of global path optimization, and it is easy to fall into local optimization. The BA is used for global path planning, which is integrated with the dynamic window method and takes the local dynamic obstacle avoidance into account.

The hybrid path planning algorithm adopts the idea of divide and conquer. The algorithm is divided into two parts. When the global environment information is known, the global path planning algorithm is used to plan a global path. On this basis, the dynamic window method is activated to plan a local path to avoid obstacles.

(23) G(ν,ω)=σ(αgdist(v,ω)+βdist(ν,ω)+γvel(ν,ω))

gdist(ν,ω) represents the distance between the current robot position and the nearest global path node. This evaluation function enables the dynamic window approach to plan a local path based on the global path, thereby ensuring the optimal path.

The basic process of the hybrid path planning method is as follows:

Step 1. Building a grid map based on the robot workspace.
Step 2. Using the improved bat algorithm to get a global shortest path without collisions.
Step 3. Extracting turning points from the global path. These turning points will be used as local targets in turn. In the improved dynamic window method, the distance and azimuth from current position to local target will be calculated.
Step 4. When the robot is moving towards the position of the local target point, once a new obstacle appears in the environment, the position information of the obstacle will be added in the hybrid path planning algorithm.
Step 5. When the robot successfully bypasses the obstacle, it will return to the original path. If the current local target point is reached or it is found occupied by an obstacle, the local target point will be switched to be the next turning point.
Step 6. Path planning and moving of the robot stops when the mobile robot reaches the end of the global path, or the end is surrounded by obstacles and the robot cannot reach it.

**Figure 1:**
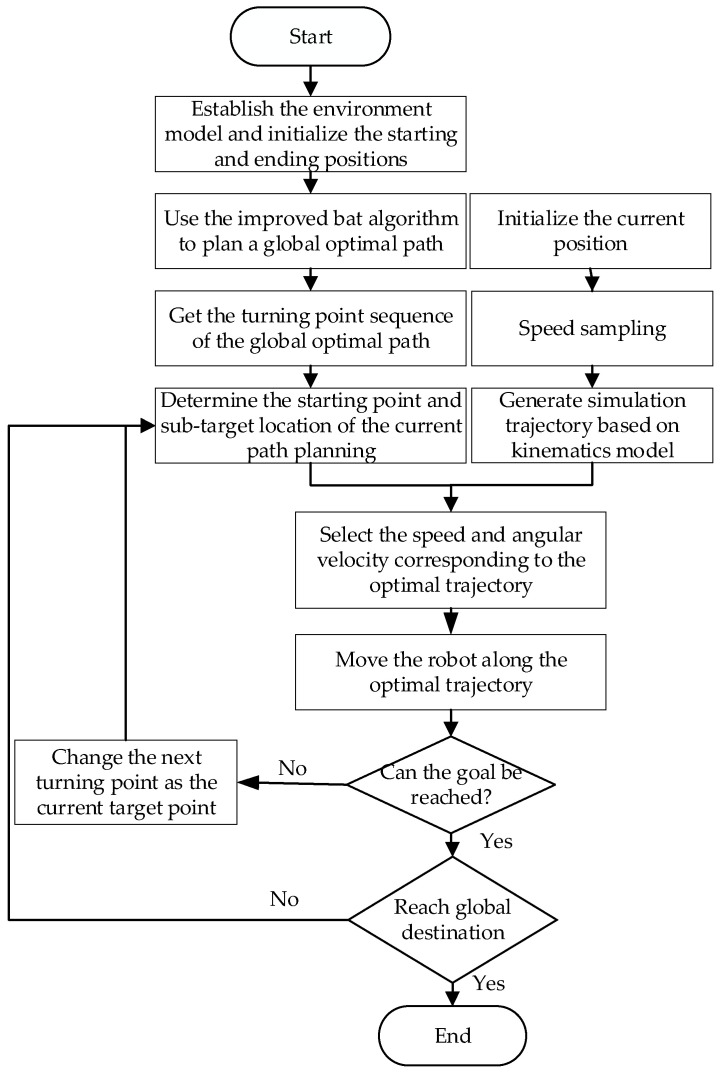
Flow chart of hybrid algorithm.

Flow chart of the hybrid path planning algorithm is shown as Figure 1:

### 4.2. Path Switch Strategy

**Figure 2:**
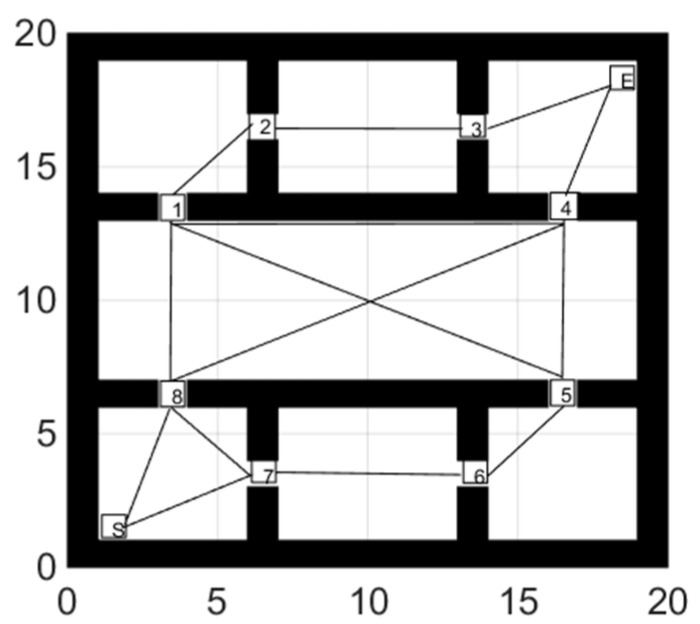
Virtual target points with the connection relationship.

In a dynamic environment, the pre-planned global path may fail. In this condition, re-planning the global path can consume a lot of time. To solve this problem, this article introduces virtual points and selects multiple virtual points (marked from 1 to 8 in Figure 2) in the environment. We add the starting point and the ending point to the figure and determine the connection between all 10 points. While planning the global path, plan the path between the virtual points with the connection relationship. Record the path and length to form an undirected weighted graph, as shown in Figure 2.

**Figure 3:**
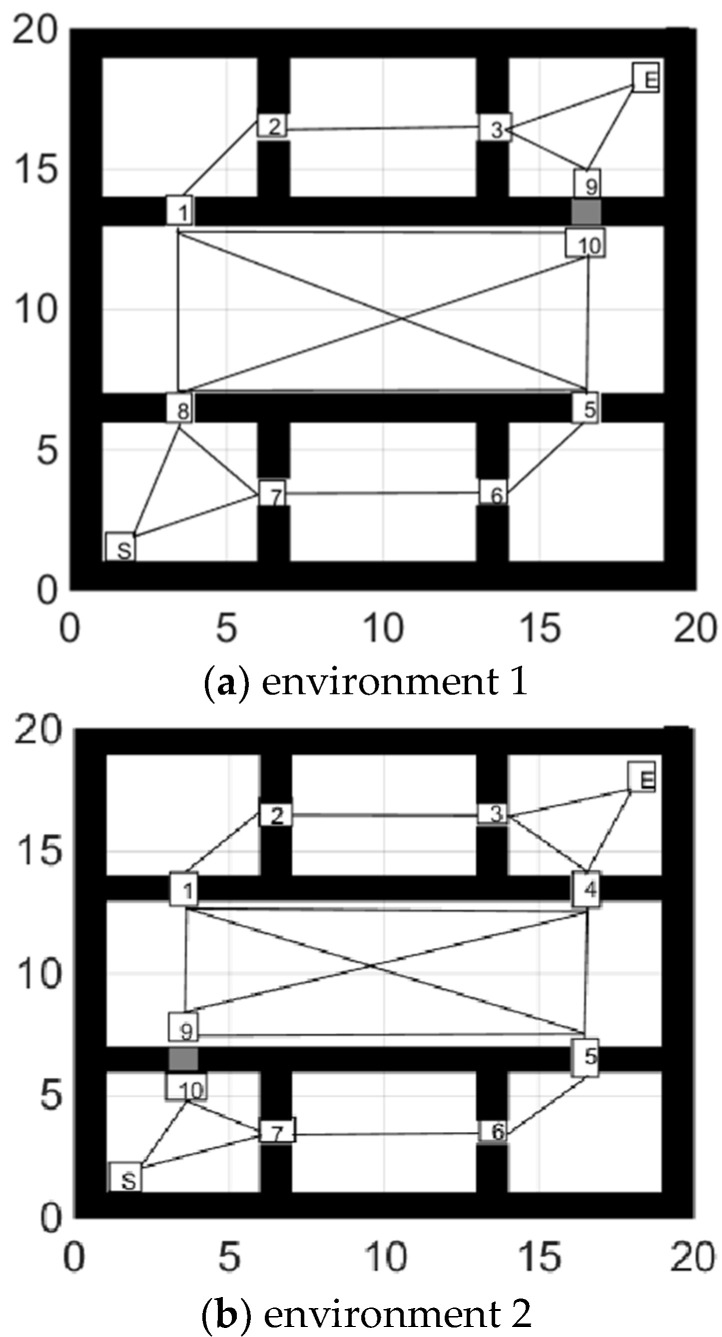
(**a**,**b**) show that an obstacle appeared at the 4th and 8th virtual points respectively.

When an obstacle appears at or near the virtual point and causes the global path to fail, we split the virtual point into multiple sub-virtual points. Sub-virtual points inherit the previous connection relationship and the path after deleting the obstacle position, as shown in Figure 3a,b. The gray grids are random obstacles. S stands for the starting point, and E stands for the ending point.

When global path planning fails, the algorithm selects the virtual node closest to the robot as the starting node and use the Dijstra algorithm to find the shortest path through the target point. The new path consists of virtual target points and is used as global path in turn to guide the robot to the target point.

## 5. Simulation Results

### 5.1. Global Path Planning

In this paper, MATLAB is used for simulation experiments to verify the effectiveness of the algorithm. First, the grid map (25 m × 25 m) is used to model the environmental information, and the global path planning is performed through the BA (Bat algorithm), IBA (Improved BA), and PSO (Particle Swarm Algorithm). In the simulation experiment, the population size of 3 algorithms is set to 50, and the maximum number of iterations is 100.

(1) Shortest collision free path

**Figure 4:**
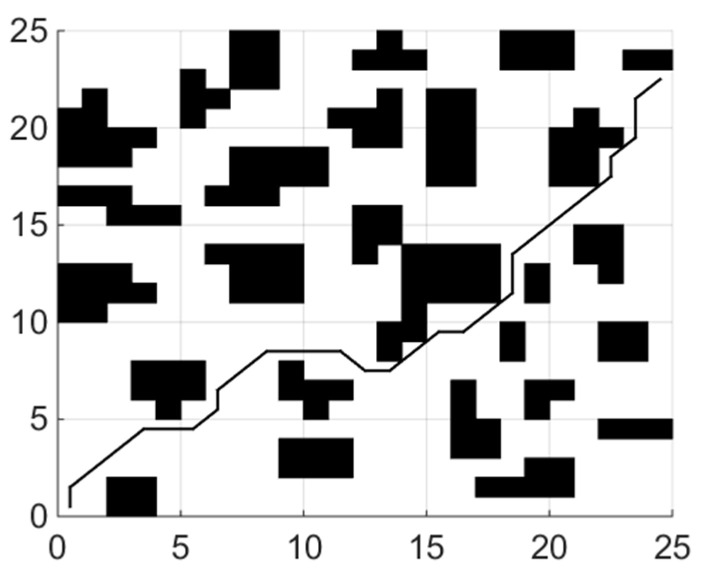
Path planning using PSO.

**Figure 5:**
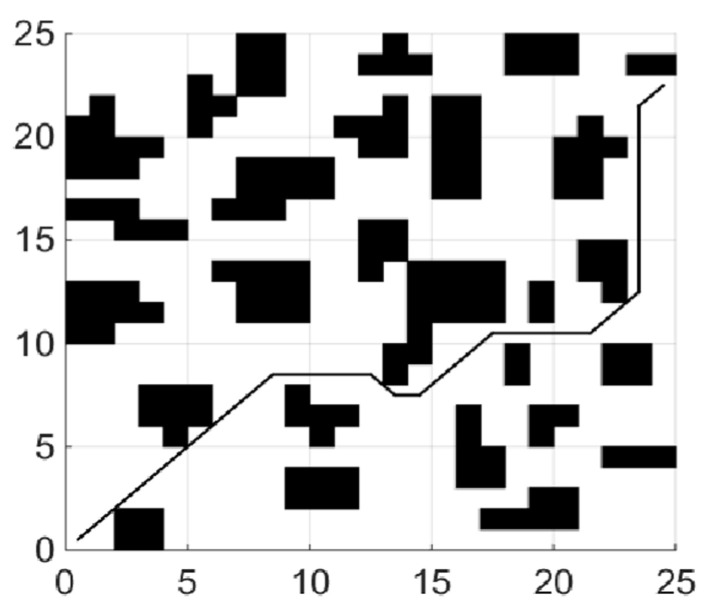
Path planning using BA.

**Figure 6:**
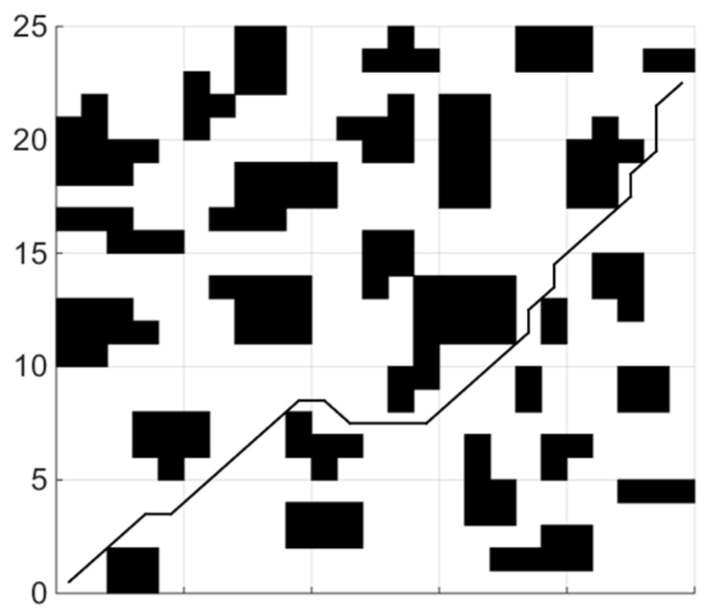
Path planning using IBA.

When the coefficient μ of the smoothing function in the fitness function is 0, the planning task is transformed into the shortest collision-free path. The experiments of each global path planning method are shown in Figure 4, Figure 5 and Figure 6. Table 1 shows length of paths obtained by each of the three algorithms running 20 times.

By analyzing the results of 20 simulation experiments, paths found by the IBA are better than the other two algorithms in term of length. The longest path found by IBA is 4.25 shorter than that of PSO, which is reduced by 9.35%, and is shorter than that of BA by 4, which is reduced by 8.85%; In aspect of the shortest path length, the result is a reduction of 3.08% and 1.58%, respectively. The average length is reduced by 7.13% and 2.88%, respectively, compared with PSO and the basic BA. In addition, variance statistics show that the performance of the IBA algorithm is more stable.

(2) Minimum number of corners

Only considering the length of the path is not enough, and we know too many corners do not only bring difficulty in robot control, but also lead to frequent acceleration and deceleration of the robot, consuming more energy. Therefore, this section comprehensively considers the path performance evaluation index by adding the path smoothness function to the fitness function. The weights of the three terms in the fitness function are η=1,λ=1,μ=0.1, and other parameters in the algorithm remain unchanged. After running the algorithm for 10 times, the statistical results are shown in Table 2.

According to the simulation results, after adding the smoothness function, the minimum, maximum and average turns of the path are reduced by 3, 4 and 3.5 times, respectively, which greatly improves the smoothness of the path and is more conducive to the control of the robot. However, adding a smoothness function will affect the path length to a certain extent.

**Figure 7:**
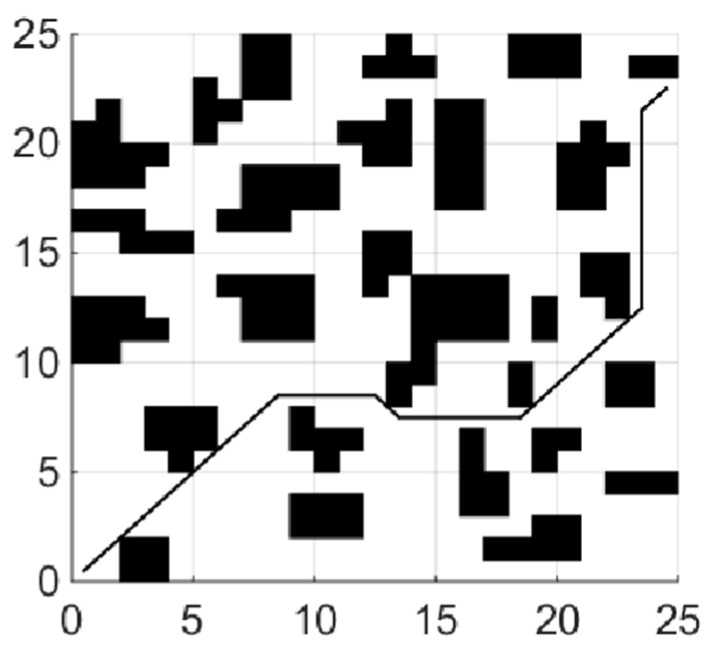
Path with least corner.

Figure 7 shows the path with the least number of corners searched by adding a smoothness function. The number of corners is 6, and the path length is 39.21. Compared with the optimal path found by the improved bat algorithm in the previous section, the number of turns is reduced by 8, but the corresponding path length is increased by 2.34 or 4.70%. The simulation results show that the smoothing function can play a great role in the optimization of the path, but it will also affect the length of the path. This means that the weight of the path smoothness function needs to be adjusted to reasonably balance the path smoothness and length in the application.

### 5.2. Hybrid Path Planning

The dynamic window approach parameters are set as follows: the maximum linear velocity is 0.5 m/s, the maximum angular velocity is 30 rad/s, the maximum linear acceleration is 0.2 m/s^2^, the maximum angular acceleration is 50 rad/s^2^, the time resolution is 0.1 s, and the evaluation function parameters are α=0.05, β=0.5, γ=0.1.

**Figure 8:**
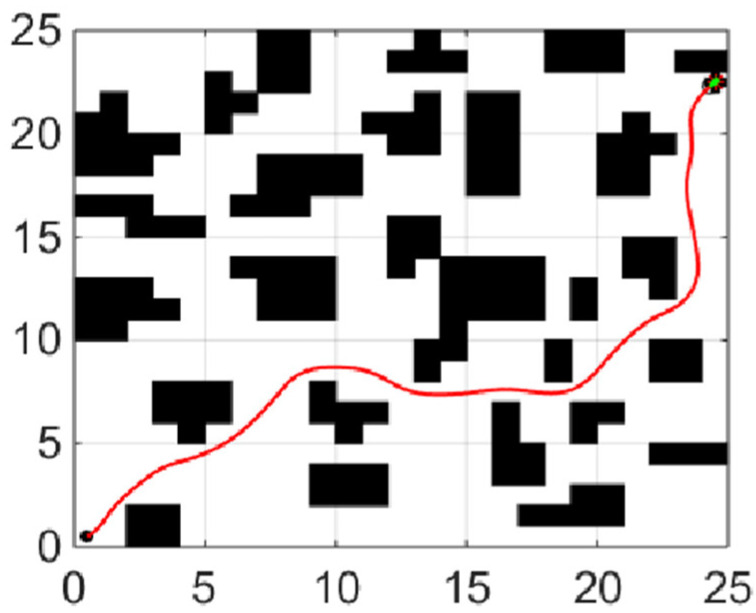
Hybrid path planning.

**Figure 9:**
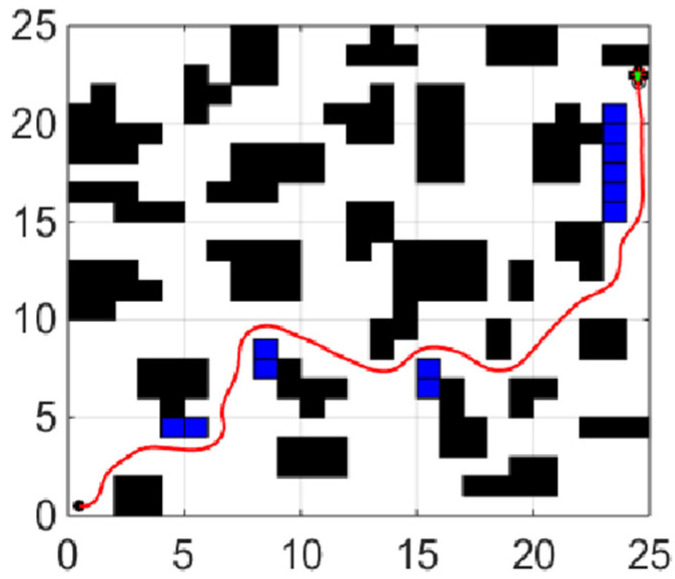
Hybrid path planning to avoid obstacles.

Figure 8 shows the experiment of the fusion algorithm proposed in this paper. The path length of the fusion algorithm is 39.23 m. Adding obstacles to the grid map we test the ability of the hybrid algorithm to avoid static obstacles. In Figure 9, the blue grid in the figure represents the newly added obstacles, and the red line represents the walking path of the robot. The dynamic window method planned a local path for the mobile robot. After successfully bypassing the newly added obstacles, it continued to move along the global path until it reached the ending point.

### 5.3. Path Switch Strategy

**Figure 10:**
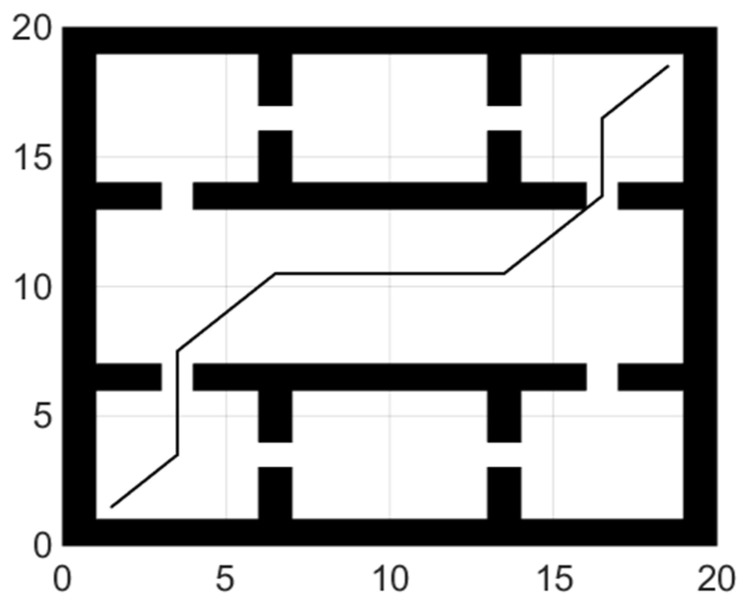
Global Path.

The global path is shown in Figure 10.

**Figure 11:**
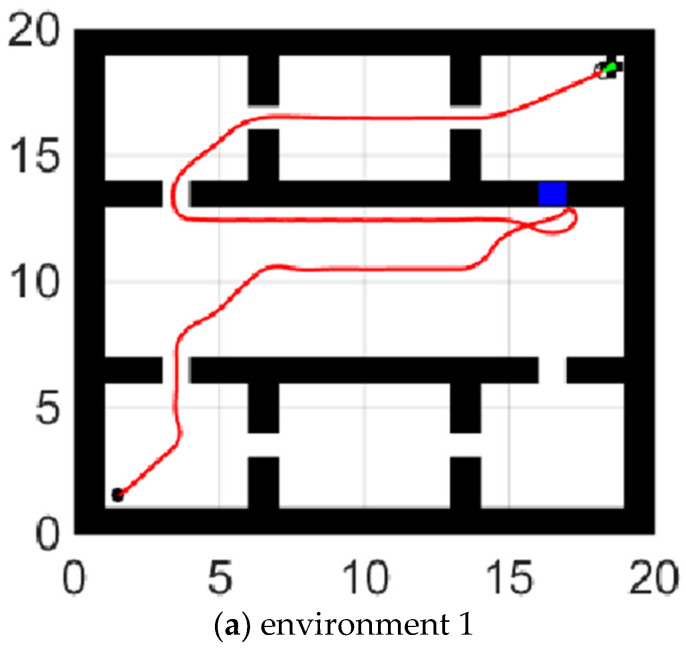

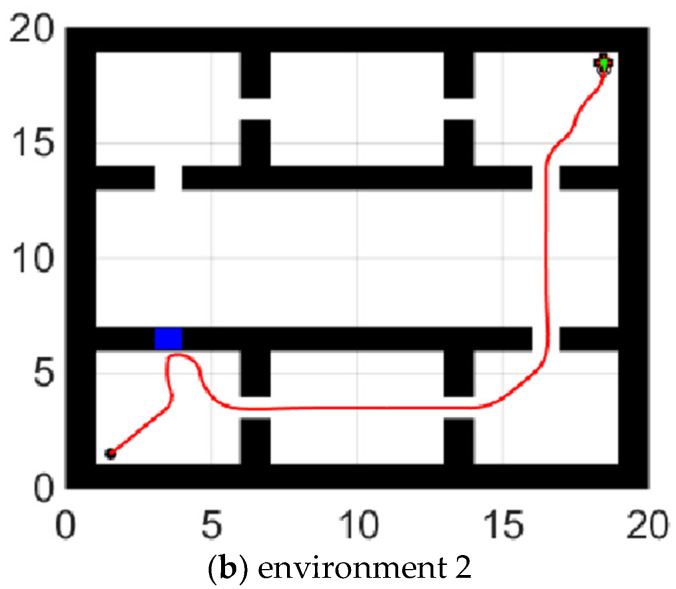
(**a**,**b**) show the robot’s path switch strategy while facing the situations in Figure 3.

The path switch strategy is shown in the Figure 11. When the global path failed, the algorithm found the alternate path and completed the path switch. In Figure 11a, the robot found that it cannot go through (17,13), and then it turned to track a new global path to the ending point. The shortest path goes through virtual node 10, 1, 2, 3, and E in turn. Similarly, in Figure 11b, the robot could not go through (3,7), and then it turned to track the path which goes through virtual nodes 7, 6, 5, 4, and E in turn.

## 6. Conclusions

In this paper, BA and DWA are combined to realize robot path planning. We use logarithmic decreasing strategy and Cauchy disturbance to enhances the searching ability of BA. The length and smoothness of the path are taken as the evaluation indexes to make the planned path smoother. In DWA, the evaluation function is designed to realize the combination of global path planning and local path planning so that DWA can carry out local path planning along the global path. The hybrid algorithm can guarantee that the planned path is better than that of the traditional DWA. The improved DWA taking both dynamic obstacle avoidance of the robot and global path tracking into account makes the robot’s movements in complex environments more efficient. In the last, we developed a new path switch strategy to reduce re-planning time. Simulation results have demonstrated the effectiveness of this method in a room space marked with virtual points.

However, this article does not conduct an in-depth study of various coefficients, and some path evaluation indicators such as energy consumption are not considered. These issues will be considered and improved in the follow-up research.

## Figures and Tables

**Table 1:** Comparison of length of paths.

Algorithm	Maximum/m	Minimum/m	Average/m	Variance/m
PSO	45.46	38.04	41.79	3.06
BA	45.21	37.46	39.96	4.65
IBA	41.21	36.87	38.81	1.81

**Table 2:** Comparison of the number of corners.

Value of γ	Maximum	Minimum	Average
0	16	9	12.6
0.1	12	6	9.1

## Data Availability

Not applicable.
